# A method for characterizing daily physiology from widely used wearables

**DOI:** 10.1016/j.crmeth.2021.100058

**Published:** 2021-07-29

**Authors:** Clark Bowman, Yitong Huang, Olivia J. Walch, Yu Fang, Elena Frank, Jonathan Tyler, Caleb Mayer, Christopher Stockbridge, Cathy Goldstein, Srijan Sen, Daniel B. Forger

**Affiliations:** 1 Department of Mathematics and Statistics, Hamilton College, Clinton, NY, USA; 2 Department of Mathematics, Dartmouth College, Hanover, NH, USA; 3 Department of Neurology, University of Michigan, Ann Arbor, MI, USA; 4 Molecular and Behavioral Neuroscience Institute, University of Michigan, Ann Arbor, MI, USA; 5 Department of Mathematics, University of Michigan, Ann Arbor, MI, USA; 6 LSA Technology Services, University of Michigan, Ann Arbor, MI, USA; 7 Department of Computational Medicine and Bioinformatics, Michigan Institute for Data Science, University of Michigan, Ann Arbor, MI, USA; 8 Lead contact

## Abstract

Millions of wearable-device users record their heart rate (HR) and activity. We introduce a statistical method to extract and track six key physiological parameters from these data, including an underlying circadian rhythm in HR (CRHR), the direct effects of activity, and the effects of meals, posture, and stress through hormones like cortisol. We test our method on over 130,000 days of real-world data from medical interns on rotating shifts, showing that CRHR dynamics are distinct from those of sleep-wake or physical activity patterns and vary greatly among individuals. Our method also estimates a personalized phase-response curve of CRHR to activity for each individual, representing a passive and personalized determination of how human circadian timekeeping continually changes due to real-world stimuli. We implement our method in the “Social Rhythms” iPhone and Android app, which anonymously collects data from wearable-device users and provides analysis based on our method.

## In brief

Bowman et al. describe a statistical method to extract a circadian rhythm in HR and other physiological parameters from real-world wearable-device data. The method fits personalized parameters of circadian timekeeping and is tested on real data from medical interns, finding consistent circadian rhythms distinct from activity and sleep timing.

## Graphical Abstract

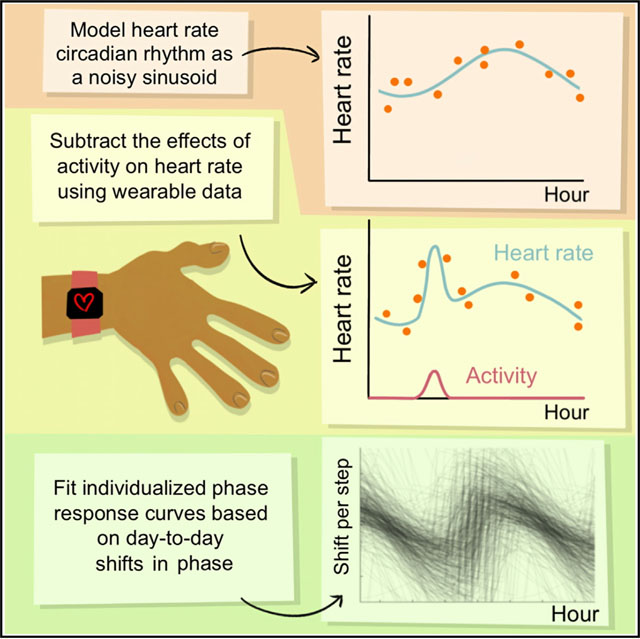

## INTRODUCTION

Measurements of heart rate (HR) have become ubiquitous with the rise of wearables. Many physical processes affect HR, notably including the circadian rhythm, an internal clock synchronizing physiological functions that has wide-ranging connections to human health. This clock can be tracked by markers such as dim-light melatonin onset (DLMO), the time at which secretion of the sleep-regulating hormone melatonin begins, measured from blood or saliva samples; however, such methods are impractical for large epidemiological studies and rarely used in clinical practice, given limited availability and lack of insurance reimbursement. Passive, easy-to-use methods to assess circadian timekeeping, e.g., using wearable-device data, are needed for population-level studies and to evaluate circadian timekeeping in real-world situations, to improve schedule design to maximize the performance and health of shift workers, and for the future of chronomedicine (Arendt, 2010; Crowley et al., 2004; Sahar and Sassone-Corsi, 2009; Walch et al., 2016).

We focus on a rich dataset from an ongoing study of medical interns (NeCamp et al., 2020). The Intern Health Study includes over 130,000 days’ worth of data from more than 900 interns who continuously wore wrist-based sleep-tracking devices collecting motion and HR data for one year. Participants working both day and night shifts provide data at all possible phases of circadian misalignment. Demographic information about this cohort is shown in Figure S1.

We use a simple statistical model to study the daily dynamics of HR in this dataset. Our model subtracts the effect of activity (Brown and Czeisler, 1992) and discards data obtained during sleep, which affects HR (Kleitman and Kleitman, 1953; Timmerman et al., 1959). We identify both an underlying circadian rhythm in HR (CRHR) and a process accounting for short-term dynamics in HR, such as those regulated by cortisol and other hormonal signals, relaying information on posture, meals, stress, and other externalities.

We show that: (1) many properties of the HR rhythms we extract from wearable devices match those of the rhythms measured in a constant routine protocol, wherein meals, posture activity, and sleep are carefully controlled; (2) the CRHR is shifted by, but does not directly track, cues such as light and sleep, like other measurements of the human circadian pacemaker; and (3) our method performs similarly across different devices, including the Apple Watch, Fitbit, and Mi Band. We additionally provide the “Social Rhythms” iPhone and Android app, which anonymously collects data from users, performs our analysis, and returns a report detailing changes in the circadian rhythm and other daily HR parameters over time.

## RESULTS

### Extracting physiological parameters from heart rate data

As a baseline, we assume a 24-h background oscillation in HR with unknown mean, amplitude, and phase (the CRHR). HR increases from this baseline proportionate to activity (steps), matching existing data (Scheer et al., 2010). Because this effect varies on the basis of physiology, fitness, and other factors, we fit a linear HR-per-step effect of activity separately for each individual. For robustness, we remove data during sleep and short interruptions (less than 2 h) in the middle of longer periods of sleep. This yields a final model for HR at hour *t* during wakefulness:

$$HR_{t}=a-b\cdot \cos\left(\frac{\pi }{12}(t-c)\right)+d\cdot \;Activity\;+\varepsilon_{t},$$

where *a* is the basal HR in beats per minute (bpm), *b* is the amplitude of a 24-h circadian oscillation in HR (which might be 0 if such an oscillation does not exist), *c* is the time of the circadian HR minimum (i.e., circadian phase), *d* is the increase in HR per unit activity (steps), and ε_*t*_ is the model error.

The error ε_*t*_ should include two known effects. First, optical HR measurements from devices worn on the wrist have limited accuracy (Wang et al., 2017). Second, many external factors affect HR: on the hour timescale, HR is affected by cortisol and other hormones (Becker and Rohleder, 2019), which are in turn driven by a range of stimuli, including prolonged standing (Liu et al., 2010; MacWilliam, 1933), meals (Fagan et al., 1986; Sauder et al., 2012), awakening (Smyth et al., 2015), light (Jung et al., 2010), and stress (Buckert et al., 2014). These effects can be increased by caffeine intake (Lovallo et al., 2006), and can vary with gender, meal content and size, type and severity of stress, and postural change. Exercise by itself does not increase cortisol (Lovallo et al., 2006), so these effects are distinct from the direct effect of cardiac demand modeled by the parameter *d*.

To account for these effects, we assume the noise ε_*t*_ at time *t* follows a common statistical error model known as an AR(1) process:

$$\varepsilon_{t+1}=k\varepsilon_{t}+N\left(0,\;\sigma^{2}\right),$$

i.e., the noise at time *t* + 1 carries over a fraction *k* of the noise at time *t* (representing the ongoing effects of external factors on HR) plus independent Gaussian noise with standard deviation *σ* (representing measurement error and new external effects). This yields six parameters (basal HR in *a*, circadian oscillation in *b* and *c*, activity in *d*, measurement error in *σ*, and other dynamics in *k*), which must be fit directly from the data. In practice, we find that *σ* and *k* are consistent across both days and individuals, corresponding to a measurement error of roughly ±15 bpm and a correlated error process on the timescale of approximately 1 h, respectively. The six parameters of our model are the simplest possible way to account for the properties we have discussed.

We average HR and steps data into 5-min bins, and section the data into “days” (periods of wakefulness that are separated by periods of sleep more than 2 h in length). We then fit our model to 2-day intervals centered at the period of sleep in between, allowing direct comparison between our phase parameter *c* and the sleep midpoint.

We fit our parameters by using Goodman and Weare’s affine-invariant Markov chain Monte Carlo algorithm (Goodman and Weare, 2010), a likelihood-based approach using approximate sampling that provides error estimates and is not affected by large gaps in data, such as when devices are charged, which can bias other approaches such as least squares (Huang et al., 2019a). The error estimates, which account for the likelihood that a particular set of parameter values matches the data, are a key part and a major advantage of our method.

**Figure 1.**
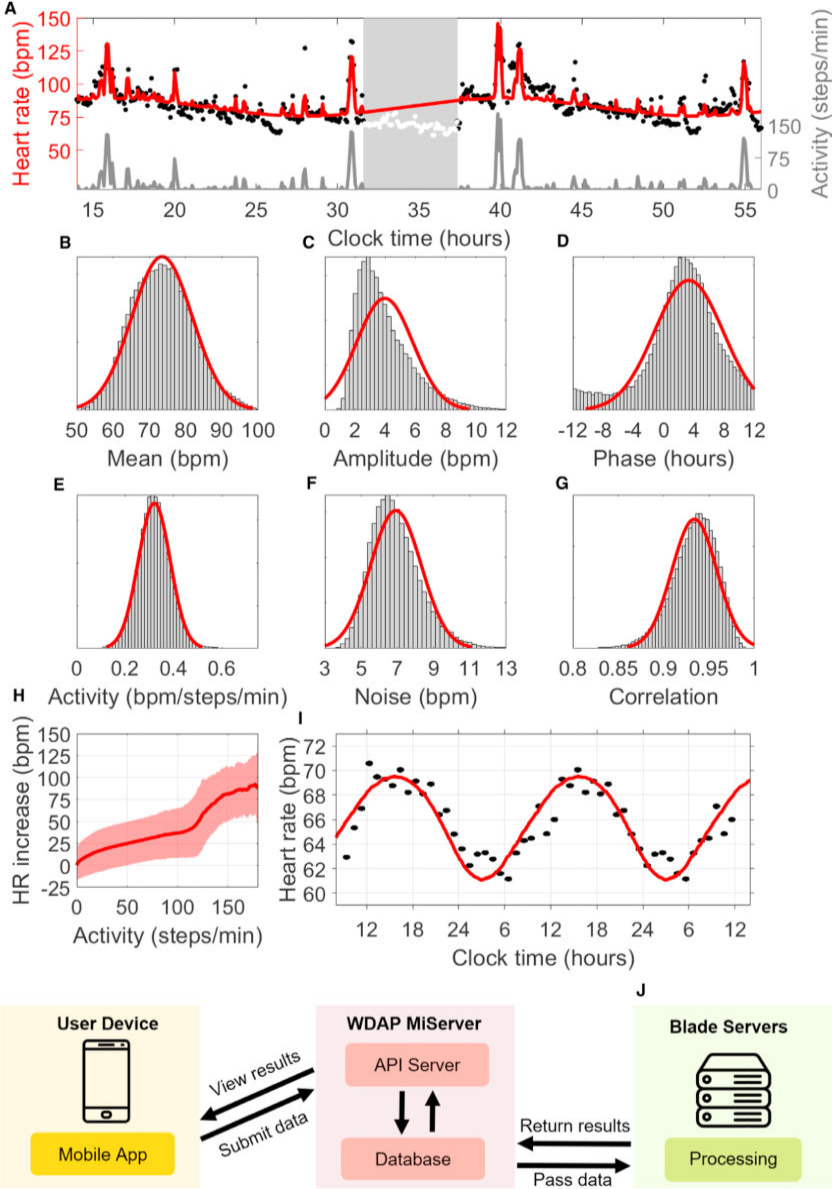
Extracting parameters from wearable data (A) Model fit (red curve) for 2 days of HR (black dots) and activity (gray curve) from a medical intern on a night shift. Times are shown in 24-h time (e.g., 36 is 12:00 p.m. on the second day). Days are separated by a period of sleep (white dots) centered at 10:29 a.m.; the HR clock phase minimum at 4:00 a.m. (±70 min) indicates desynchrony (CRHR phase is advanced compared with the sleep-wake cycle). (B) Fitted mean HR for 2-day periods centered at 133,775 recorded instances of sleep (μ = 73.5214, σ = 8.3588 bpm), with normal fit (red) for visualization only. (C) Fitted HR amplitude (μ = 3.9605, σ = 1.8609 bpm). (D) Fitted HR phase (μ = 3.3311, σ = 4.5492 h). (E) Fitted effect of activity (μ = 0.3205, σ = 0.0660 bpm/steps/min). (F) Fitted independent noise level (μ = 6.9159, σ = 1.3937 bpm). (G) Fitted noise correlation between consecutive minutes (μ = 0.9339, σ = 0.0247). (H) Average increase in HR at different levels of activity (solid curve) with 95% prediction bands (shaded region). (I) Comparison of 24-h background oscillation in HR estimated by our model (red) with the average HR measured by G. Vandewalle et al. for n = 8 healthy males in a constant routine (black) (Vandewalle et al., 2007). (J) Schematic of the Wearable Data Analysis Platform (WDAP), which analyzes user-submitted data.

Figure 1A shows the model fit for a 2-day period from a medical intern; the red curve tracks a background 24-h rhythmicity plus the predicted effect of measured activity. (Given that we do not use sleep data, nor do we attempt to model the effect of sleep on HR, the red curve shown during sleep serves only to help visualize the phase and amplitude of the oscillation and is unrelated to HR measurements during that period.) This individual was working a night shift and slept from approximately 7:30 a.m. to 1:30 p.m., but their CRHR was consistent with a normal sleep schedule. Like other circadian clocks, the CRHR can be out of sync with the sleep-wake cycle.

In Figures 1B–1G, we show the fitted parameter values for 136,789 days of Fitbit data obtained from 927 individuals in the Intern Health Study (Sen et al., 2010). All possible circadian phase estimates *c* were occasionally observed, as we studied a population of shift workers. Circadian amplitudes *b* were typically between 1 and 6 bpm. Although other parameters exhibit variation between days and individuals, all fell within a range of typical values. Basal HR *a* was usually between 65 and 85 bpm. The noise parameter *k*, which appears in Figure 1G, consistently yielded a correlation time of roughly 1 h, matching the known dynamics of cortisol. The steps-to-HR parameter, shown in Figure 1E, consistently measured around 0.3, i.e., increasing activity level by 1 step per minute elevated HR by roughly 0.3 bpm. Small deviations in these parameters could be used in future studies to identify and characterize illness. Parameters were similar across age groups, although our cohort had a narrow age distribution, and no significant gender differences were seen (Figure S1, bottom).

To keep computational costs at a manageable level, parameters were fitted by testing only 100,000 probabilistically chosen possible fits (“samples”) for each day of data. Increasing the number of samples for a random subset of 5% of the data did not significantly affect parameter estimates, but did yield a modest reduction in error estimates (Figure S2), and circadian phase had a statistical uncertainty of ±1 h when using 200,000 samples or more. When the computational cost is not as significant, the number of samples could be increased accordingly.

We now show that the statistical components of Figures 1B–1G correspond to true physiological properties. Figure 1H shows a direct comparison of HR to activity across all individuals. Although there is significant variation between individuals (shaded region), which we account for by fitting subjects separately, a consistent upward trend is seen in the raw data. Figure 1E shows that the estimated values of *d*, our model parameter fitting a linear effect of activity on HR, closely match the slope of this trend. The shape in Figure 1H matches the experimental literature, including a bend for intermediate activity levels (Tudor-Locke et al., 2019). More complex models were found to have little influence on the estimated values of other parameters; the linear approximation via the parameter *d* captures this relationship well.

Subtracting out the estimated effect of activity and averaging across all subjects reveals an average 24-h rhythmicity in HR, shown in Figure 1I, determined by parameters *b* and *c* of our model. Vandewalle et al. measured a similar curve in an experiment under a constant routine protocol, a clinical protocol wherein activity, sleep, and other factors were carefully controlled (Vandewalle et al., 2007). Our estimates from non-clinical data closely match the amplitude and phase observed by Vandewalle et al., suggesting that our model’s circadian component is capturing a true physical process. (The mean HRs differ significantly, due to major differences between the constant routine protocol and our real-world data and differences between age and athletic ability of subjects, etc. Previous work has shown that changes in mean HR do not affect the amplitude or phase of circadian oscillation in HR (Scheer et al., 2010).)

To facilitate the future analysis of data from a wide range of settings, we created a Wearable Data Analysis Platform (WDAP, Figure 1J), which collects and processes data anonymously submitted by the public through the Social Rhythms app, available for free through the App Store (iOS) and the Google Play Store (Android). An application program interface server transmits anonymous user-submitted data to a processing server, which analyzes the data by using our method. When analysis is complete, users view results through the app.

### Real-world dynamics of the circadian rhythm in heart rate

Our statistical model extracts physiological parameters for activity, circadian effects, measurement noise, and short-term dynamics. Notably, we consistently find a 24-h circadian signal in HR in all subjects, which validates against clinical results obtained in constant routine; that this circadian effect could be isolated from HR data in an uncontrolled setting was not obvious. This provides a unique opportunity to study the dynamics of this CRHR in a real-world setting.

**Figure 2.**
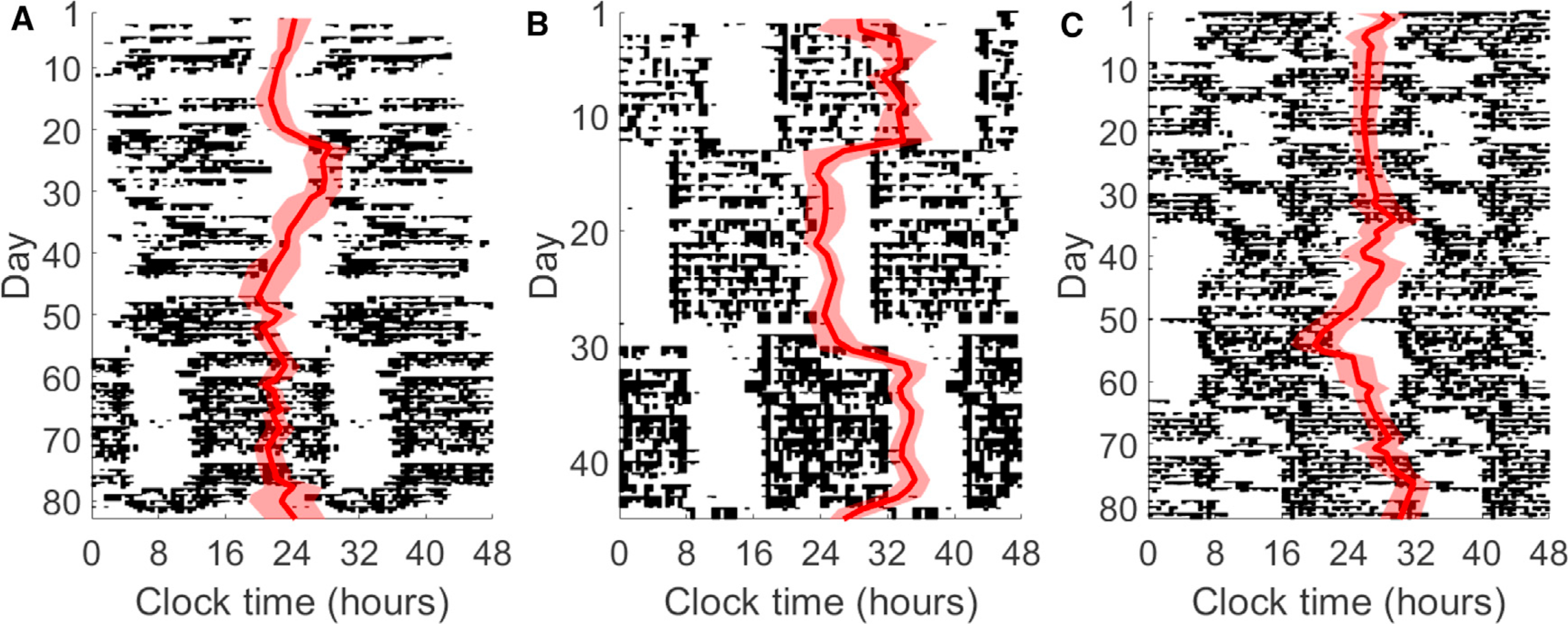
Dynamics of CRHR in real-world data (A–C) Actograms for three subjects in the Intern Health Study generated from Fitbit data. Estimated CRHR phase (red, with 80% confidence bands) is overlaid on daily activity patterns (black). Shown in (A), subject A maintains a consistent CRHR throughout a shifted sleep schedule, whereas (B) subject B quickly adjusts. In (C), subject C exhibits two distinct responses to different shifts in activity.

Figure 2 shows the circadian phase parameter *c,* the circadian minimum of HR, tracked over a period of 2–3 months for 3 individuals from our dataset (see GitHub repository for more examples). The phase estimates (red line) with 80% confidence bands (shaded region) are overlaid on actograms, which use black histogram-like bars to show measured activity patterns throughout the day. When daily routines are consistent, CRHR phase tracks with sleep.

Major disruptions in daily routine yield a range of behaviors in CRHR. All three individuals transition through one or more periods of shift work, when the activity pattern shifts by several hours between days. In days 1–50, intern A (Figure 2A) slowly adjusts to a later schedule, and the CRHR follows. A new shift begins around day 55, yielding a dramatic 7-h shift in activity pattern; however, the HR clock does not shift, instead following other consistent cues rather than sleep timing, such as activity, light exposure, or meals. In contrast, intern B (Figure 2B) adjusts to new shifts quickly, and the estimated circadian phase swung dramatically over 3–4 days to match the new sleep schedule. Intern C (Figure 2C) shows that different responses might be observed within one individual, and the circadian signal in HR remained consistently out of phase for the first shift (days 10–35) but gradually adjusted later on. Shift workers use different strategies to adjust to new schedules; this is, to our knowledge, the first large-scale continuous measurement of a circadian marker during these adjustment periods.

Two authors of this study recorded additional data from an Apple Watch (1,071 days) and a Mi Band (189 days); phase estimates and actograms for these data appear in the supplemental information (Figure S3). The Apple Watch author simultaneously wore a Fitbit for approximately 200 of the 1,071 days; phases estimated from the two devices were strongly correlated (p = 0.00096). Both Fitbit and Apple Watch data suggested that, for this author, CRHR remained consistent through a period of international travel around day 90, which resulted in a major shift in activity patterns.

### Relationship between CRHR and sleep

**Figure 3.**
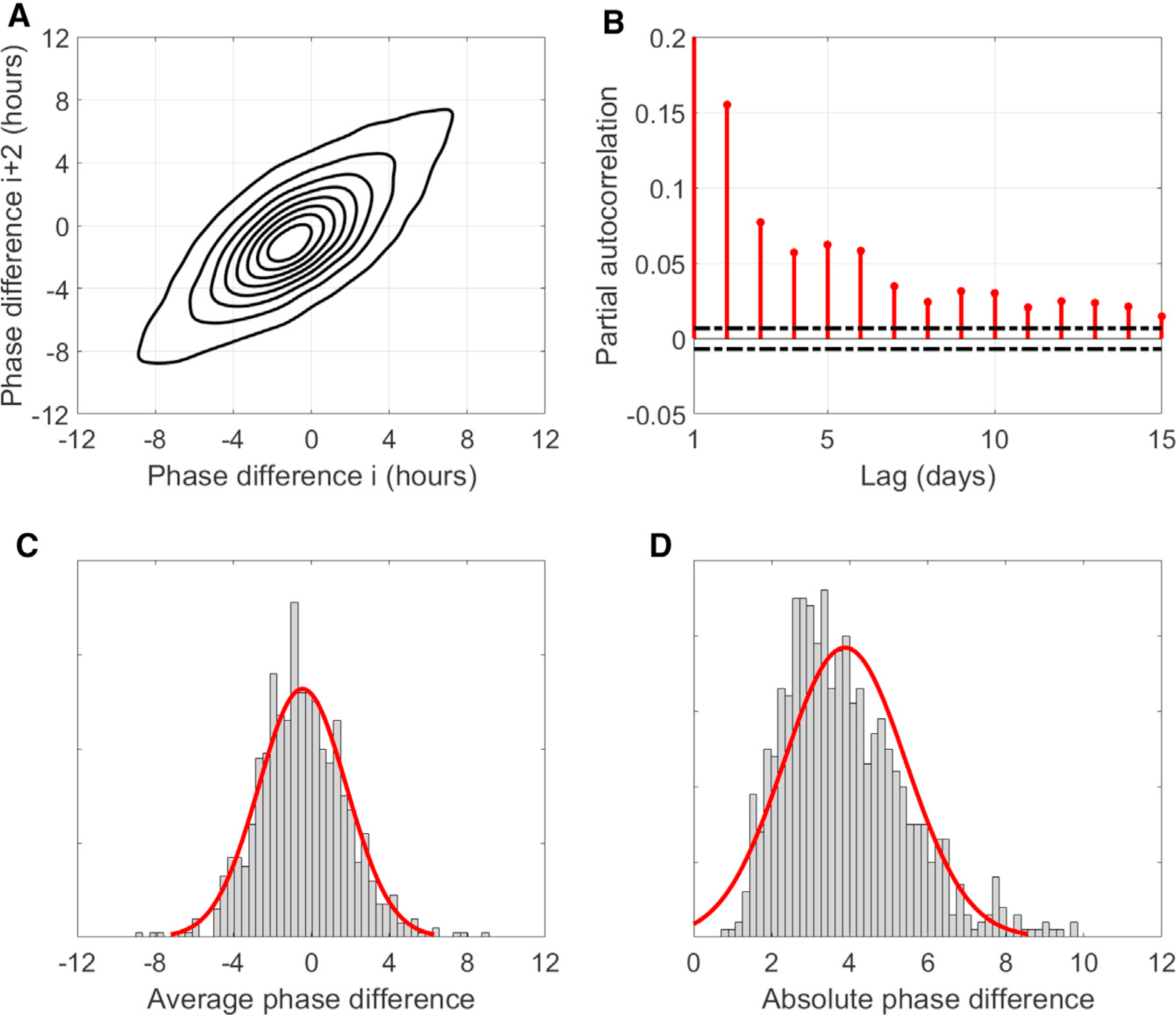
Analysis of phase difference between midsleep and CRHR (A) Contour-plot comparison of phase differences between CRHR phase and sleep midpoint over a 2-day period for n = 133,775 estimated phase differences from 927 individuals in the Intern Health Study. When the CRHR is out of phase with sleep, it is likely to be similarly out of phase in 2 days. (B) Partial autocorrelation function of phase differences; the correlation between phase differences separated by a certain lag in days (red lines) remains statistically significant (outside black dotted lines) out to roughly 1–2 weeks. (C) Histogram of average phase difference across all subjects (μ = −0.45, σ = 2.25 h) with normal fit (red) for visualization only. (D) Histogram of average absolute phase difference across all subjects (μ = 3.88, σ = 1.56 h), which includes errors due to uncertainty and shifting sleep schedules, with normal fit (red) for visualization only.

Figure 2 shows that the CRHR acts independently and can be driven significantly out of phase from the sleep cycle. Given that all data obtained during sleep were discarded, this result cannot be affected by any effect of sleep on HR. We define the “phase difference” as the difference in hours between estimated HR phase—the circadian minimum of HR—and the sleep midpoint (as reported by the device), e.g., a phase difference of 4 h would indicate that HR is at its lowest 4 h after the midpoint of sleep. Figure 3A compares each night’s phase difference to the same individual’s phase difference 2 nights later. On average, phase difference is close to zero (HR mostly aligns with sleep), but if the CRHR is out of phase with sleep, it usually remains that way 2 nights later. Using the partial autocorrelation function, a statistical tool for looking at correlations over time, Figure 3B performs a similar comparison up to 15 days into the future. The correlation (red) remains statistically significant (outside dotted black lines) out to 2 weeks in the future, although the magnitude of the correlation falls on the order of 1 week. The 24-h rhythm in HR tends to synchronize with sleep only gradually over several days (or sometimes not at all).

Figures 3C and 3D show the frequency of average phase differences in the Intern Health Study. The range of values we observe is larger than one would expect from other circadian signals like DLMO, which are generally more closely aligned with the sleep-wake cycle (Lewy et al., 1999). For most individuals, this is the average of hundreds of days of data; it follows that this phase marker is likely affected by factors other than those that influence DLMO. The average absolute difference (Figure 3D), which averages only the magnitude and not the sign of the phase difference, corroborates this result, although it also incorporates errors due to uncertainty and shifts in the sleep schedule (which might temporarily drive the HR clock out of phase with sleep, even if they are usually synchronous).

### A personalized phase-response curve of CRHR

Circadian clocks are often characterized by using phase-response curves (PRCs), which measure how the clock responds differently to a stimulus at different times of the day (Khalsa et al., 2003). This is a defining feature of circadian timekeeping: the ability to respond differently at different times of the day strongly suggests the presence of a self-sustained circadian clock. Such a curve therefore serves as a test of the inherent timekeeping ability of the CRHR. PRCs can be generated for a range of stimuli (most commonly, bright light); here, we use activity, measured by the wearable device via steps. We previously removed the acute effects of activity on HR, which occurs on the timescale of minutes, and now look for the more subtle effect of how activity could phase shift the circadian rhythm itself, which would be seen on a much longer timescale of days.

**Figure 4.**
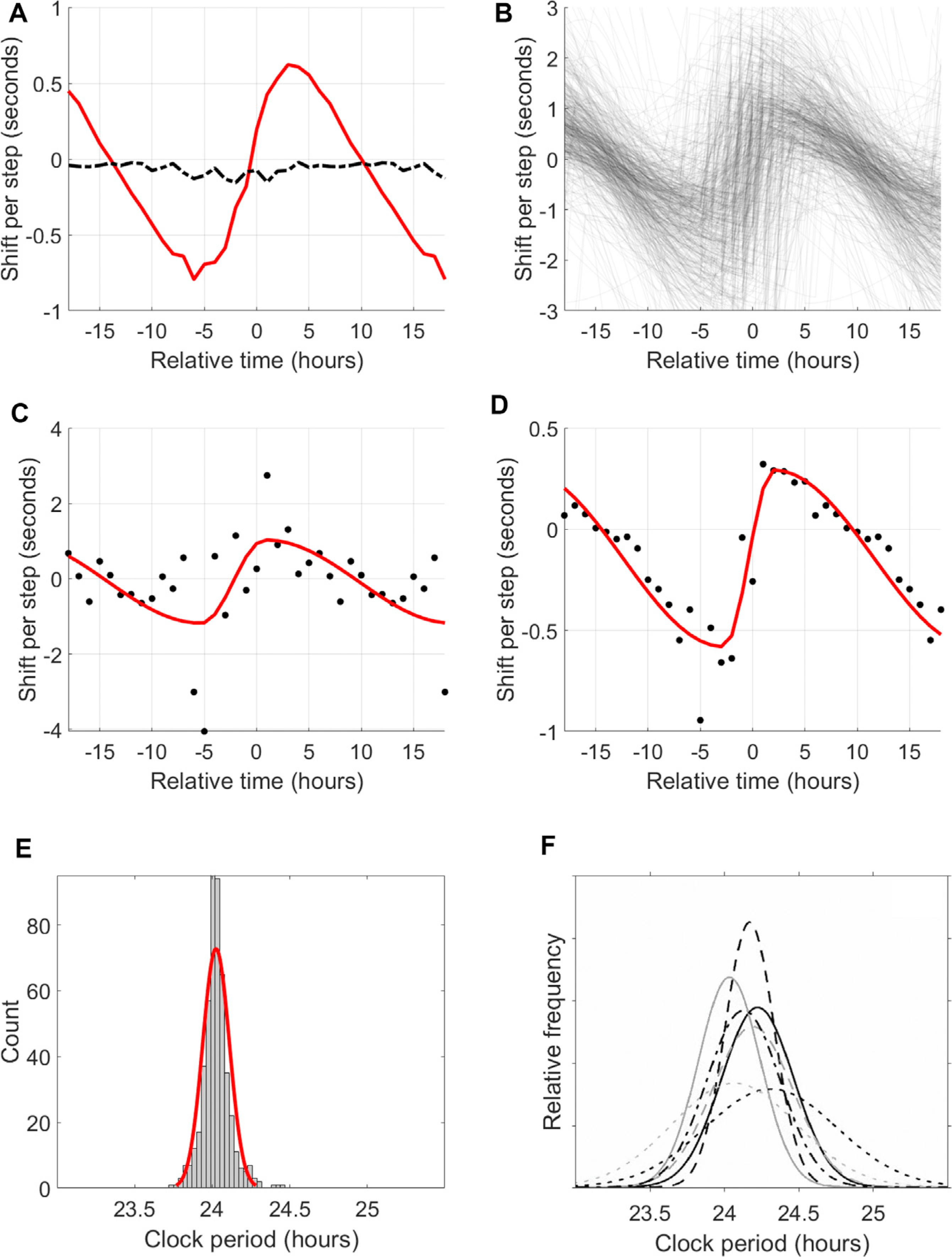
Phase-response curves of CRHR to activity (A) Average phase-response curve (red) for all subjects with at least 50 days of data. Relative time is hours before/after CRHR minimum (e.g., 5 means 5 h after circadian minimum, usually in the morning); shift per step is the average phase-shifting effect of one step at that time (by convention, negative shifts correspond to phase delays and positive shifts correspond to phase advances). When hours of activity are randomly shuffled, the relationship vanishes (dashed black line). For ease of visualization, some points are double-plotted, extending the 24-h curve to a 36-h plot. (B) Cloud of individual phase-response curves for 524 Intern Health Study subjects. (C) Raw PRC data (black, 1-h bins) with parameterized fit (red) for the Mi Band dataset (189 days). (D) Raw PRC data with parameterized fit for the Apple Watch dataset (1,071 days). (E) Histogram of the CRHR periods suggested by the vertical offset of each PRC (μ = 24.03, σ = 0.08 h), with normal fit (red) for visualization only. (F) Reproduced distributions of periods of circadian clocks found by forced desynchrony studies (Woelders et al., 2017).

We hypothesized that activity (and related factors, such as light exposure, which correlate with measured activity) would be a suitable proxy for the signals that entrain the HR clock; recent modeling work suggests that activity measurements can outperform even light measurements in predicting DLMO (Huang et al., 2019b). To characterize this effect, we calculated a PRC for each individual (see STAR Methods) to activity. We found a robust PRC for basically all individuals (Figure 4).

As a first comparison, we calculated the average PRC for all individuals with at least 50 days of data (Figure 4A). The result is similar in shape to the PRC to light previously measured for humans (Khalsa et al., 2003), including a non-zero vertical offset, which has previously been linked with the non-24-h intrinsic period of the clock. It also matches a PRC to activity where effects on melatonin were quantified (Youngstedt et al., 2019). These comparisons suggest that the primary entrainment signal for the CRHR is activity, acting in a way similar to how light signals melatonin.

We also generated individual PRCs for each subject (Figures 4B–4D). Because many tens or hundreds of consecutive daily measurements of phase are needed to generate these curves, this would be impractical using DLMO techniques. These PRCs are completely personalized—they do not use data from other subjects or from the average “human clock”—and yield consistent shapes across individuals and across devices.

Some characteristics of these individual PRCs have physical interpretations. For example, human circadian clocks are known to have a period slightly longer than 24 h (Czeisler et al., 1999; Hiddinga et al., 1997), so each PRC should have a small vertical offset corresponding to the difference between the true periodicity and the fixed 24-h period of the component we extract. Figure 4E shows a histogram of the resulting estimates of the true CRHR period (μ = 24.03, σ = 0.08 h). We reproduce in Figure 4F the distributions of circadian periods found by a range of clinical studies (Woelders et al., 2017); our distribution is in line with this family of distributions, although with somewhat smaller spread. This could be due to the relatively narrow demographics of our study (medical interns ages 20–30) or to inherent differences between the CRHR and other circadian clocks.

The distributions of three other characteristics of these curves appear in the supplemental information (Figure S4). The sinusoidality of the curve (Figure S4A) is related to the coupling of individual cellular oscillators within the suprachiasmatic nucleus (SCN) (Hannay et al., 2015). The amplitude (Figure S4B) may reflect conditioning to activity; for example, athletes might have clocks that are less shifted by activity than sedentary individuals, as their metabolic demands are different. Finally, the horizontal shift (Figure S4C) varied among individuals but was similar to the spread observed between sleep midpoint and HR clock phase (Figure 3C). Demographic factors such as age and gender were not found to have significant effects on the PRC.

## DISCUSSION

We have shown that daily physiological parameters, such as basal HR and HR circadian phase, can be passively assessed by using HR and activity measurements from common wearable devices. We have purposefully checked our method against several possible confounders to HR. For example, could the CRHR that we observe reflect patterns of physical activity? First, we specifically removed the effects of activity through our statistical model. Second, our estimate of CRHR does not follow activity, as when activity timekeeping shifts in individuals, CRHR often does not. Could CRHR reflect sleep-wake patterns? Again, the timing of the CRHR is distinct from the timing of sleep. Figure 3 shows that differences between midsleep and CRHR have temporal dynamics on the scale of days to weeks and that a mean difference between CRHR and midsleep, measured for more than a year for many subjects, varies between individuals.

What about other factors, such as stress, medications, salt intake, etc., which affect HR? Could disease states influence these markers? First, it is important to note that the correlated noise process in our statistical model represents many possible external effects on HR, most prominently those regulated by hormones such as cortisol. Second, we show that the phase and amplitude of the rhythms we measure across our population match closely those found in a constant routine protocol, a protocol that is explicitly designed to control such effects. Our ability to capture this circadian signal from real-world data is a major benefit for population-level studies, although a carefully designed clinical study could also attempt to control these effects directly. We note that even clinical studies using the constant routine protocol suffer from effects such as sleep deprivation, and other authors have raised questions about using methods so dissimilar to real life, particularly when measuring HR (Kerkhof et al., 1998; Duffy and Dijk, 2002). That our method is built on real-world data, which implicitly includes these external factors, is a selling point, for example, to diagnose disease (by looking for changes in HR physiology) in a wide population.

That PRCs (Figure 4) can be generated by using long-term measurements from wearable devices is a remarkable result. Traditional PRC protocols are extensive, are costly, and must pool data from many (>20) subjects; each data point is the result of an individual living in a time isolation clinic for several days to a week. Over the course of hundreds of days, with timescales achieved only with wearables, we gain the statistical power to look at activity at any given time of day and correlate it to phase shifts in CRHR between one day and the next. This characterization of an individual’s circadian clock is important to a range of applications in personalized medicine.

Although DLMO is often considered a gold standard marker of circadian phase, many have questioned its role in validating real-world circadian markers. Phase estimates using core body temperature (CBT), another widely used marker, correlate only loosely with estimates from DLMO, with a correlation coefficient of less than 0.5 (Komarzynski et al., 2019). High-carbohydrate meals might shift CRHR and CBT, but not DLMO (Krauchi et al., 2002). Even melatonin onset and offset show different dynamics (Liu and Borjigin, 2005), raising questions about which parts of the melatonin profile are most appropriate to compare against (Phillips et al., 2019). In animal models simulating jet lag, autonomous circadian clocks in the heart and other peripheral organs shift at different rates compared with the central clock located in the SCN (Damiola et al., 2000; Stokkan et al., 2001). All of this suggests that CRHR, as a distinct circadian marker, need not have the same characteristics as other markers such as DLMO.

For normally entrained individuals, we find that CRHR keeps a constant relationship with sleep, and would therefore generally agree with DLMO. However, we find several features of the rhythm in HR that are different from what DLMO has predicted. First, the average phase relationship between our marker and sleep has more interindividual variation than is found with DLMO. This suggests that DLMO might act in the body to better predict sleep, especially because of melatonin’s effects on sleep, whereas CRHR might time other factors. We see very similar phase responses by the CRHR to activity and by DLMO to light, suggesting activity might be the main entraining signal for HR rhythms. We also see that activity has a greater ability to generate long-lasting phase shifts in CRHR than in DLMO. Finally, the period of the CRHR clock might be more tightly regulated than that measured by DLMO (compare Figures 4E and 4F).

These results are exactly in line with physiology. Recently, the source of the circadian variation in HR was discovered to be in the sinoatrial (SA) node of the heart, rather than the central circadian pacemaker in the SCN, which controls DLMO (D’Souza et al., 2020). One would then expect that activity would be a larger signal than light for the CRHR, especially as the SCN has direct input from the retina. The electrical activity of the SA node is tightly coupled, whereas the SCN is known to be less tightly coupled (DeWoskin et al., 2015). Moreover, specific mechanisms within the SCN allow for period adjustment by photoperiod. Thus, we would reasonably expect that the period of the SCN would show more interindividual differentiation than that of CRHR. Having the same overall PRC shape is likely due to the conserved mechanisms of molecular timekeeping, including having the same molecular input pathway. HR can be affected by sleep deprivation, whereas DLMO typically is not, which suggests its role in regulating sleep (Holmes et al., 2002).

Understanding HR is vitally important because it is a critical marker of increased risk of cardiovascular disease. Our work clarifies the expected characteristics of HR on a daily timescale, including an underlying circadian rhythm and the effects of activity, potentially leading to more accurate measures of what constitutes a normal or abnormal HR. Our Social Rhythms app and online platform have already been tested by ~2,000 unique users, opening the possibility of future population-based studies of physiology predicted by daily patterns in HR.

### Limitations of study

The effects of caffeine, psychological stress, disease, pharmaceuticals, etc., on HR are not directly accounted for in our parameter estimates, but could be explicitly included in future work. Certain types of cardiovascular activity, such as weightlifting and bicycle riding, might affect HR in ways that are not accounted for by the way wearables report activity; use of raw motion data should be investigated. Sleep as measured by wearables (used only to discard data during sleep) is not as accurate as that measured in the lab. Proprietary algorithms might use time-of-day information to score sleep, although non-proprietary algorithms exist (Walch et al., 2019). (Our algorithms do not directly depend on sleep scoring; sleep scoring is used only to remove sleep data that might bias our estimates.) Finally, we observe deviations from the linear relationship between steps and HR, especially as individuals transition from walking to running. The importance of these differences in measuring our six physiological parameters (if any) should be further investigated.

## STAR⋆METHODS

### RESOURCE AVAILABILITY

#### Lead contact

Further information and requests for resources should be directed to and will be fulfilled when possible by the lead contact, Daniel B. Forger (forger@umich.edu).

#### Materials availability

No physical materials were created in association with this study.

#### Data and code availability

All code used in this study can be found in a publicly available Github repository at https://github.com/pepperhuang/heartrate. The main algorithm is called from the MATLAB file main.m. Two data sets are provided: 1235 heartrate and steps include the author’s Apple Watch data, while 100012 HR, Sleep_Stage, and Step include the author’s Fitbit data.

Any additional information required to implement the method of this study is available from the lead contact upon request.

### METHOD DETAILS

We estimate CRHR phase using Bayesian uncertainty quantification. To ensure that periods of more frequent measurement do not disproportionately affect results, heart rate and activity data are averaged into five-minute bins. The Fitbit and Mi Band data sets also include sleep timing, which we use to discard all data obtained during sleep (the Apple Watch data set already had sleep removed, as the watch was charged overnight). Wakeful periods of less than two hours in the middle of a period of sleep are also discarded. Data from pairs of consecutive days (centered by a period of sleep) are fit to a 24-hour sinusoidal model with three parameters (phase, mean, amplitude) plus a linear effect from activity using a scaling parameter. Together with two parameters (correlation, noise) describing the autoregressive AR(1) error model, six parameters are sampled from the likelihood using Markov chain Monte Carlo (MCMC). When predicting phase on successive days, the previous day’s fit (plus Gaussian noise with s.d. 1 hour) is used as a prior distribution, and the posterior is sampled instead of the likelihood. Phase estimates (means) and uncertainties are calculated directly from this weighted cloud of fits.

Phase response curves are calculated in 24 one-hour bins. For each day of data, we first shift activity by the CRHR phase estimate from the previous night, so that the timing of activity is when the activity occurred relative to the current circadian phase estimate, rather than relative to clock time. (Activity occurring at time 0 would be exactly when heart rate is at its minimum – usually during sleep.) We then bin activity into one-hour bins, with, e.g., the first bin corresponding to all activity that occurred between 0 and 1 hours after CRHR minimum. The total amount of activity in that bin becomes a value of x; the phase difference between the previous night and the following night is the corresponding value of y, and so the point (x,y) describes that for one day with x activity at a certain relative time, we observed a phase shift of y. We record the point (x,y) and to which bin it corresponds. After recording observations for all days of data, each bin contains a large number of points (x,y) – one for each day of data. Simple linear regression is used to estimate the average slope within each bin, which will be the average phase shift per step for all activity during that bin (e.g., between 0 and 1 hour after circadian minimum).

Since our phase estimates used two days’ worth of data (so that intervals are centered at a period of sleep and can be compared to sleep timing), one concern was that the phase difference from one night to the following night would be biased from being fitted to overlapping data (the day of heart rate data in between was used in both phase estimates). We separately generated PRCs using phase changes separate by two or more, which did not have any data in common. As results were almost identical, we kept the simplest comparison (phase change over 24 hours). When plotting a smooth curve fitted to our 24 one-hour bins, we used nonlinear least-squares to fit a sinusoidal model with three parameters (phase, mean, amplitude) plus a fourth parameter (trough-to-peak time) allowing the first and second half-periods to have different lengths.

### QUANTIFICATION AND STATISTICAL ANALYSIS

The lines denoting statistical significance in Figure 3 are calculated using the standard method for the full and partial autocorrelation functions in the MATLAB routines *autocorr* and *parcorr*.

The strength of correlation referenced in Figure S3 used a simple linear regression comparing phases estimated using Apple Watch data with phases estimated using Fitbit data. The stated p-value is the p-value of a hypothesis test for significance in slope.

### ADDITIONAL RESOURCES

Intern Health Study portal: https://www.srijan-sen-lab.com/intern-health-study

## Supplementary Material

1

2

## Figures and Tables

**KEY RESOURCES TABLE:** 

REAGENT or RESOURCE	SOURCE	IDENTIFIER

Deposited data		

Author Fitbit, Apple Watch, Mi Band data	This paper	https://github.com/pepperhuang/heartrate

Software and algorithms		

Social Rhythms App	This paper	https://apps.apple.com/us/app/social-rhythms/id1510826025
MATLAB	The MathWorks Inc.	https://www.mathworks.com/
Bayes Circadian Phase Code	This paper	https://github.com/pepperhuang/heartrate

Other		

Intern Health Study	A prospective cohort study investigating factors associated with depression during medical internship.	https://pubmed.ncbi.nlm.nih.gov/20368500/

## Highlights

- Statistical method tracks six physiological parameters from wearable heart-rate data
- Method is tested on 130,000+ days of heart-rate data from medical interns
- Results show a circadian rhythm in HR consistent with clinical data
- Method provides personalized phase-response curves of HR to activity

## MOTIVATION

The exploding popularity of wearable devices, now a multi-billion dollar industry, provides a new opportunity for real-world data collection. Here, we propose a statistical method for analysis of ambulatory wearable-device data that can estimate circadian rhythms. Accounting for circadian rhythms in HR will allow more accurate measurement of other physiological parameters, e.g., basal HR, how activity increases HR, and changes in HR due to infection.
