# Supplementary material for: A method for characterizing daily physiology from widely used wearables
Source: Cell Rep Methods. 2021 Jul 29;1(4):100058. doi: 10.1016/j.crmeth.2021.100058 (PMC8462795; doi:10.1016/j.crmeth.2021.100058)
Supplement: Document S1. Figures S1–S4 [file mmc1.pdf]

Cell Reports Methods, Volume *1*

Supplemental information

A method for characterizing daily physiology from widely used wearables

Clark Bowman, Yitong Huang, Olivia J. Walch, Yu Fang, Elena Frank, Jonathan Tyler, Caleb Mayer, Christopher Stockbridge, Cathy Goldstein, Srijan Sen, and Daniel B. Forger

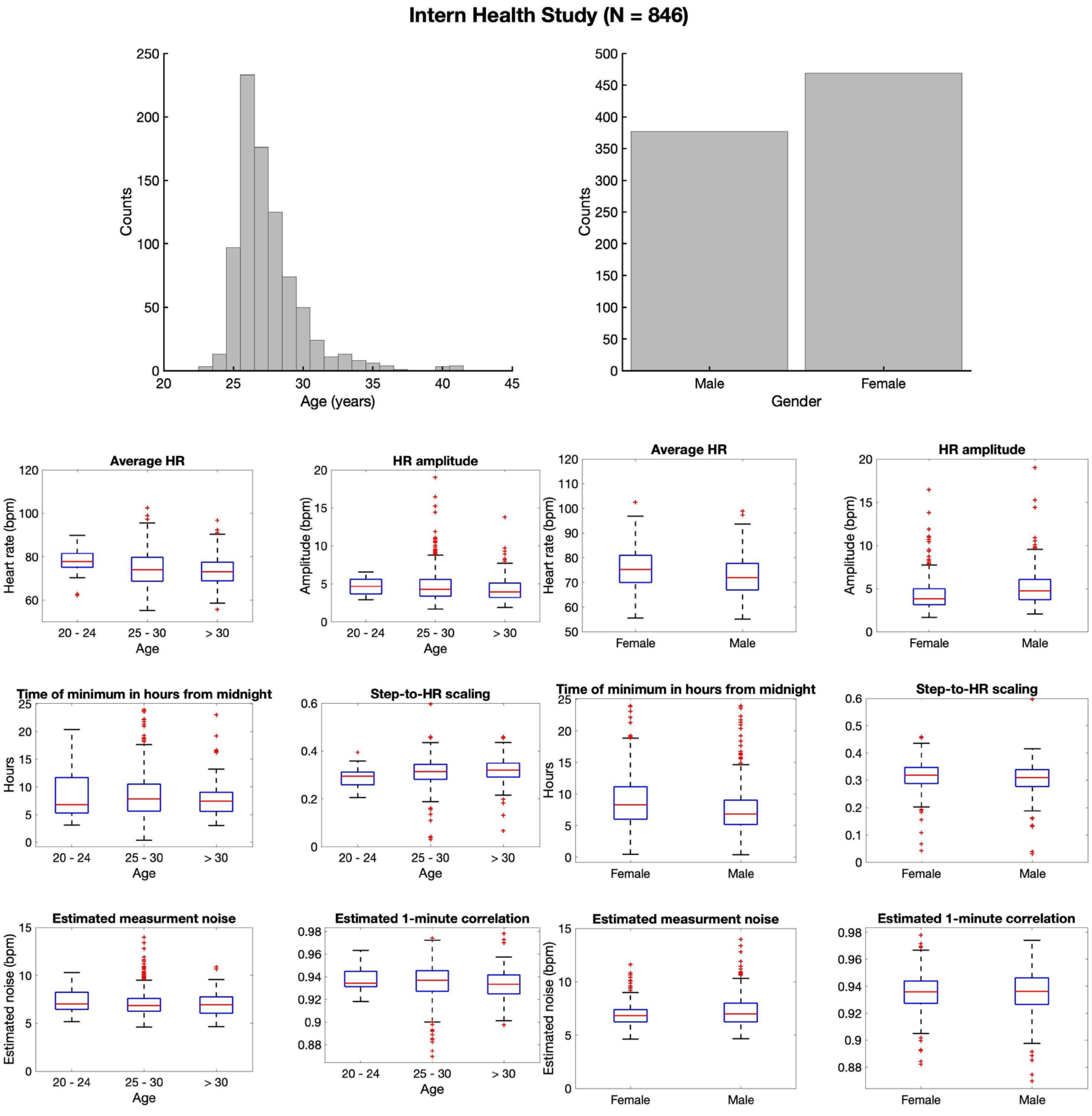

Figure S1. Demographic information and parameter comparison for the 2018-2019 Intern Health Study, related to Figure 1. (Top) Interns are evenly distributed between genders and largely fall between 25 and 30. (Bottom) Differences in CRHR model parameters (*a, b, c, d, k,* σ) between age groups and genders. No significant differences were observed, i.e., circadian rhythmicity in heart rate did not show significant correlation with age or gender.

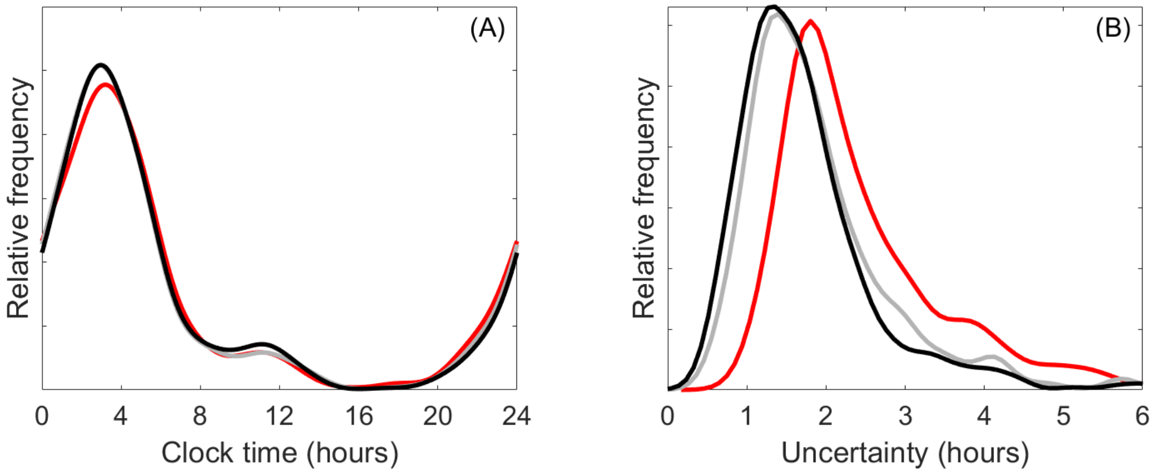

Figure S2. Effects of sampling on our methodology, related to Figure 1. (A) Increasing the number of samples in our parameter fitting (using GWMCMC) in a random subset of 5% of interns from 100,000 (red) to 200,000 (gray) to 500,000 (black) samples did not significantly change our estimates of phase. (B) Increasing the number of samples from 100,000 (red) to 200,000 (gray) yielded a modest reduction in the statistical uncertainty Further increase to 500,000 samples (black) had little additional benefit, suggesting 200,000 samples is sufficient even when the data set is small enough that computational cost is not a concern.

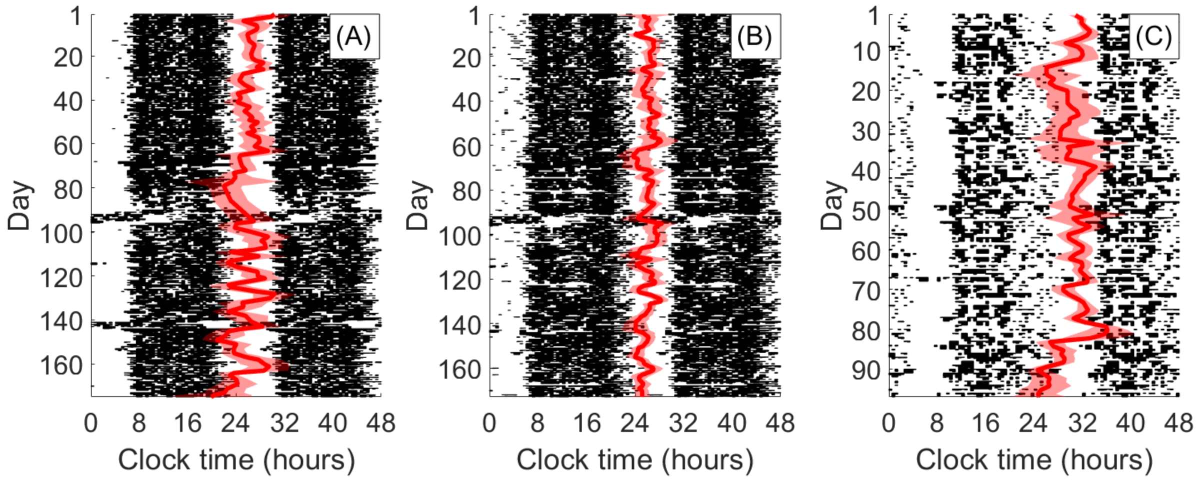

Figure S3. Testing our methodology across devices, related to Figure 2. (A, B) Comparison of estimated CRHR phase for an individual wearing both an Apple Watch (A) and a Fitbit (B) simultaneously; results are strongly correlated ($p = 0.00096$), showing that both devices measure the same underlying rhythm. This individual did not undergo shift work, but did travel twice across time zones. (C) Estimated CRHR phases for data from a Mi Band, a less expensive brand of wearable. Mi Band HR measurements resulted in wider confidence bands, which could be due to the device or indicative that this author's circadian rhythm is more difficult to measure.

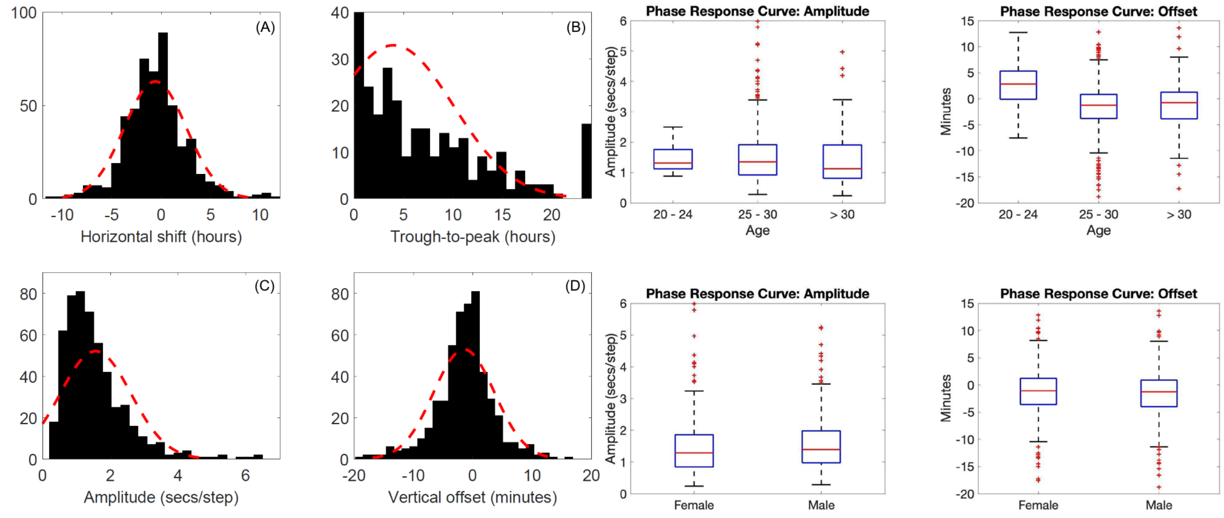

Figure S4. Personalized phase response curve characteristics and relationship to demographic information, related to Figure 4. (A) Histogram of horizontal offsets for individual PRCs (μ = -0.61, σ = 3.14 hours; Gaussian fit shown with dashed red line). Zero means the curve crosses from negative to positive exactly when baseline HR is at its lowest. (B) Histogram of trough-to-peak values (time between minimum and maximum) for individual PRCs (μ = 3.97, σ = 6.06 hours). Many individuals did not have sufficient steps at all phases to resolve this parameter well; values at 0 (bar extends upward) and 24 are half-period fits with jump discontinuities. (C) Histogram of individual PRC amplitudes (μ = 1.56, σ = 1.05 seconds per step). (D) Histogram of individual PRC vertical offsets scaled by average steps (μ = -1.48, σ = 5.14 minutes); negative values should indicate clock periods longer than 24 hours (positive values, shorter). The predicted clock period from this offset appears in Figure 4E. (Right) Summary of relationship between PRC parameters and demographic information. No significant differences in the shape of the PRC were observed when separating by either age or gender.
